# PROTOCOL: Interventions for improving executive functions in children with Fetal Alcohol Spectrum Disorder: Systematic review and meta‐analysis

**DOI:** 10.1002/cl2.1009

**Published:** 2019-07-26

**Authors:** Joseph Betts, Sharon Dawe, Elizabeth Eggins, Doug Shelton, Haydn Till, Paul Harnett, Ned Chandler‐Mather

**Affiliations:** ^1^ School of Applied Psychology Griffith University Brisbane Queensland Australia; ^2^ Gold Coast University Hospital Southport Australia; ^3^ Gold Coast Hospital and Health Service Southport Australia

## 1 BACKGROUND

### 1.1 The problem, condition or issue

Prenatal alcohol exposure (PAE) is associated with profound and lifelong disability. The umbrella term fetal alcohol spectrum disorder (FASD) describes a spectrum of impairments resulting from the deleterious effects of PAE (Chudley et al., 2005). Historically, the spectrum comprised four disorders: fetal alcohol syndrome (FAS), Partial FAS (pFAS), alcohol‐related neurodevelopmental disorder (ARND), and alcohol‐related birth defects (ARBD; Lange, Rovet, Rehm, & Popova, 2017). All four disorders shared PAE as their etiological base and all but ARBDs were associated with neurological deficits (ARBD comprizing physical defects). More recently, the Australian Guidelines to the diagnosis of FASD have been published (Bower & Elliot, 2016). While the core aspects of the condition remain unchanged, the Australian guidelines comprise two diagnostic categories for FASD: (a) FASD with three sentinel facial features or (b) FASD with less than three sentinel facial features.

While information on prevalence rates remains a significant challenge (Roozen et al., 2016), estimates have been as high as 5% in general population studies in the United States of America (USA) (May et al., 2014, 2018). Further, evidence suggests this rate may be much higher in indigenous communities (Burd, & Moffatt, 1994; Fitzpatrick et al., 2015) and the child‐protection system (Lange, Shield, Rehm, & Popova, 2013). Understanding how to support children with a diagnosis of FASD is particularly important given the condition has been linked to a range of poor outcomes, including increased contact with the justice system, substance misuse (Burd, Fast, Conry, & Williams, 2010), antisocial and delinquent behaviour, learning disabilities, externalizing, and aggressive behaviour, as well as a range of other adaptive functioning and mental health problems (Bower & Elliot, 2016; Kodituwakku, 2009; Rasmussen, Andrew, Zwaigenbaum, & Tough, 2008). While there are no agreed upon usual treatments following a diagnosis of FASD, much of the clinical literature acknowledges the important of ensuring families and caregivers have an understanding of how FASD can impact on children's behaviour and cognitions (Reid et al., 2015).

There is growing evidence that a core deficit underpinning many of these adverse outcomes is impairment in executive functions (EFs; Khoury, Milligan, & Girard, 2015; Kodituwakku, 2009). EFs are higher‐order mental processes which allow individuals to deploy attention strategically, hold and manipulate goal‐relevant information and consciously enforce goal‐directed behaviour (Baggetta & Alexander, 2016; Diamond, 2013). According to Diamond, EFs comprise three core abilities: (a) inhibitory control—the ability to inhibit prepotent responses of attention, behaviour, thoughts, and/or emotions in favour of what is appropriate or necessary; (b) working memory – the ability to hold information in mind and manipulate it in goal‐directed ways; (c) cognitive flexibility—the ability to solve problems using different perspectives or rules as they arise. Different combinations of these core EFs produce a range of higher‐order manifestations, including reasoning, problem‐solving, planning, and directing attention (Diamond, 2013).

Children with FASD have frequently shown impairment compared to typically developing children across a wide range of both core EFs and higher order manifestations (Rasmussen, 2005). Deficits have been found on cognitive inhibition, verbal and nonverbal fluency (Schonfeld, Mattson, Lang, Delis, & Riley, 2001), use of attentional strategies, planning (Green et al., 2009), visual attention, spatial working memory (Rasmussen, Soleimani & Pei, 2011), behavioural inhibition, the ability to form complex concepts and cognitive flexibility (Rasmussen et al., 2013). Research also suggests that EF impairments are often more severe than would be suggested by intelligence deficits alone (Connor, Sampson, Bookstein, Barr, & Streissguth, 2000).

Importantly, executive function deficits may underlie a number of the above mentioned poor life outcomes associated with FASD. For example, FASD is associated with a higher rate of attention deficit and hyperactivity difficulties (Rasmussen et al., 2010), and research has demonstrated that poor executive function may underpin ADHD. Poor executive functioning has also been linked to a range of other outcomes found in FASD populations, including autism (Geurts, Verté, Oosterlaan, Roeyers & Sergeant, 2004), obesity (Crescioni et al., 2011), lower quality of life (Davis, Marra, Najafzadeh, & Liu‐Ambrose, 2010), poorer school readiness (Willoughby, Magnus, Vernon‐Feagans, Blair, & Family Life Project Investigators, 2017), poorer school performance (Borella, Carretti, & Pelegrina, 2010), financial problems, criminal behaviour, and substance misuse (Moffitt et al., 2011). Consequently, addressing EF deficits in FASD populations may offer an important opportunity to significantly improve both individual and societal outcomes.

While recent years have seen an increase in studies assessing interventions designed to improve EF functioning in children with FASD, a critical gap in the literature is the absence of an updated, comprehensive systematic review and meta‐analysis in the area. Given the compromised outcomes associated with EF deficits, and the frequency of EF impairment in children with FASD, a rigorous synthesis of the effectiveness of available interventions offers great value to practitioners, individuals with FASD and their families.

### 1.2 The intervention

To be included in this review the study must use a structured psychological intervention that aims to improve or change EF in children using either (a) a face‐to‐face format, (b) computerized format, or (c) both. Interventions must be delivered using (a) individual or (b) group format. Finally, interventions will be considered eligible if they are administered either (a) directly to children (e.g., working memory training) or (b) to children and caregivers/families (e.g., the GOFaR program, described below).

Computerized training is an example of an intervention administered directly to children. This form of intervention has been used in attempts to facilitate working memory in a range of children, including pre‐schoolers (Bergman Nutley et al., 2011), intellectual under‐performers (Holmes, Gathercole, & Dunning, 2009), and children diagnosed with ADHD (Holmes et al., 2010). Recently, Kerns, MacSween, Vander and Gruppuso (2010) used computerized interventions aiming to improve attentional capability in children diagnosed with FASD. Non‐computerised cognitive training has also been used by Loomes, Rasmussen, Pei, Manji, and Andrew (2008), who employed cognitive rehearsal training aimed at improving working memory in children with FASD.

Another program that has been evaluated across multiple studies is the Alert program. Originally designed to help children with learning disabilities to utilize a number of self‐regulation strategies (Williams & Shellenberger, 1996), the Alert program uses the analogy of a car engine to teach children about self‐regulation (‘just like a car engine, our bodies can run fast or slow’). The program is typically taught in a classroom environment with weekly sessions covering a range of cognitive learning and sensory activities (Gill, Thompson‐Hodgetts, & Rasmussen, 2018). Ultimately, the Alert program aims to help children recognize their arousal states and expand self‐regulation strategies. The first randomized controlled trial of the program in children with FASD was conducted by Wells, Chasnoff, Schmidt, Telford, and Schwartz (2012). Children were randomly allocated to receive Alert or a no‐treatment control group. EF outcomes were measured using the Behaviour Rating Inventory of Executive Function (BRIEF; Gioia, Isquith, Guy & Kenworthy, 2000), a questionnaire completed by caregivers that assesses EF behaviours at home and school. Children were also given the Robert's Apperception Test for Children (Palomares, Crowley, Worchel, Olson, & Rae, 1991), which assessed the children's emotional problem solving abilities.

Nash et al. (2015) also conducted a randomized controlled trial in which children were assigned to the ALERT program or delayed treatment control group. Executive function outcomes were assessed using a variety of methods. A battery of tests was administered to children, including EF sub‐tests of the Developmental Neuropsychological Assessment (2nd edition; NEPSY‐II) (Korkman, Kirk, & Kemp, 2007), the Test of Everyday Attention for Children (TEA‐Ch) (Manly, Robertson, Anderson & Nimmo‐Smith, 1999) and the Cambridge Neuropsychological Test Automated Battery (CANTAB; Fray, Robbins and Sahakian, 1996), and parents completed the BRIEF. The Alert program was also evaluated by Soh et al. (2015) in a study design that consisted of random allocation of children diagnosed with FASD to either an immediate‐treatment or delayed control treatment group, with typically developing children serving as controls. Executive function was measured using EF sub‐tests from the NEPSY‐II, and parent report of EFs using the BRIEF. Finally, brain imaging techniques were used to map concurrent changes to brain structure.

An example of an intervention delivered to parents and children is the GOFaR program. The program is underpinned by a strategy known as FAR, an acronym standing for (a) Focus and plan (b) Act (c) Reflect (Coles, Kable, Taddeo, & Strickland, 2018). The intervention is underpinned by findings that executive function skills can be improved through direct instruction (Wells et al., 2012). Using the FAR technique, children are taught to control their attention and approach problem‐solving thoughtfully rather than relying on impulse. Children are then taught to reflect on their action in terms of what worked and what did not work relative to their goals. Most recently, Coles et al. (2018) conducted a small pilot using GOFaR to support behaviour change in children with confirmed PAE. The program used the FAR metacognitive strategy, teaching the three steps of focus (F), act (A) and reflect (R). Children and caregivers were randomized to either (a) the GoFar program (b) Faceland – an alternative program designed to improve facial expression recognition or (c) an inactive control group. Executive function outcomes were assessed using the Tests of Variables of Attention (TOVA; Greenberg & Waldmant, 1993), NEPSY‐II sub‐tests, and behavioural measures of EF were assessed using the BRIEF.

## 2 HOW THE INTERVENTION MIGHT WORK

### 2.1 Child‐centred interventions

A common intervention of this type is specific EF training. Training interventions are the intentional teaching of skills through repetitive experience aimed at restoring or improving functions and are generally completed in the presence of a qualified practitioner. These interventions can be delivered face‐to‐face (Loomes et al., 2008), or as computerized training programs (Holmes et al., 2010). Tasks generally aim to improve skills by offering practice of specific abilities unique to EF domains (Holmes et al., 2010). These interventions often involve working through levels of increasing difficulty, allowing for continual optimization of training impact (Klingberg et al., 2005). Child‐centred interventions can also be longer‐term programs which children work their way through a predefined structure over a number of sessions (See the Alert Program; Nash et al., 2015). These programs use similar mechanisms to short‐term training, relying on experiential activities to restore of improve functions.

### 2.2 Parental involvement in EF training

In addition to focusing on the development of EFs in children, a number of programs provide instruction and support to parents and caregivers. This additional component can sit alongside the EF focus and be provided either concurrently in group or individual formats to parents and caregivers or as separate program components. For example, Coles, Kable, Taddeo, and Strickland (2015) supplemented computerized training for children with FASD with a parental workshop on how PAE impacts neurodevelopment. Parents were presented with the course concurrently to their children undertaking the computer training. This aspect of intervention is often designed to improve parents’ working knowledge of their child's neurodevelopmental functioning, facilitating the provision of more effective behavioural scaffolding from parent to child (Coles et al., 2018). Scaffolding of behaviours by parents and caregivers have been shown to support the generalization of skills and provide children with an opportunity for support and repeated practice (Hammond, Müller, Carpendale, Bibok, Maximilian & Liebermann‐Finestone, 2012). Sensitivity analyses will be conducted to explore whether parental involvement in interventions alters the impact on child outcomes.

### 2.3 Why it is important to do the review

#### 2.3.1 Previous relevant publications and knowledge‐gaps

In recent years increasing awareness of the prevalence of FASD across the world has sparked new interest in trialling interventions designed to ameliorate the deleterious effects of PAE on functioning. A number of recent systematic reviews have been published in the areas of FASD prevalence (Popova, Lange, Probst, Gmel, & Rehm, 2017; Roozen et al., 2016), comorbidity (Popova et al., 2016), and to assess the impact of treatment (Peadon, Rhys‐Jones, Bower, & Elliott, 2009; Reid et al., 2015). The most recent review by Reid et al. (2015) provided a synthesis of the effectiveness of treatment interventions for FASD that included an assessment of methodological quality of FASD intervention studies across the lifespan. Thus, this narrative review included interventions targeting parenting skills, self‐regulation and attentional control, mathematics skills, non‐verbal reasoning, and social skills.

A key limitation of Reid et al.'s review is the lack of a quantitative synthesis of effect sizes. By providing a meta‐analysis in parallel to the review, more precise conclusions can be provided regarding the overall efficacy of interventions that address a core deficit in children with FASD. The current study will therefore provide both an updated review of interventions aimed at enhancing EFs and a quantitative synthesis of these studies. A secondary objective of this review is to examine whether the effectiveness of interventions vary by a range of factors, including

1. Variation in the number, setting, delivery and intensity of program components;
2. Variation in program participants (e.g. gender, age, comorbid diagnoses, level of PAE), and;
3. Variation in the type of EF targeted by the intervention.

A systematic review protocol has also recently been published by Singal et al. (2018) aiming to review effectiveness of interventions in FASD populations. The review intends to include meta‐analysis if possible, however aims to evaluate any outcome pertaining to children's physical and mental health as well as cognitive, behavioural and social skills. Whilst this review will provide a comprehensive and broad examination of the extant evaluation literature, a more focused review in a single domain affected by PAE will provide a concise synthesis of the existing evaluation studies and provide clear directions for policy and practice. By focusing on one domain of functioning (EF) and just two types of interventions common in FASD research, this review seeks to provide a more targeted synthesis of available literature. Thus, the current study will be the first to provide a single, overall measure of effect size for EF interventions in children.

#### 2.3.2 Practice and policy relevance

Globally, there have been a range of initiatives aimed at preventing FASD through increased public awareness of the effects of PAE. Concurrently, we have seen the development of clearer diagnostic processes (e.g., Astley & Clarren, 2000; Bower and Elliot, 2016; Chudley et al., 2005). This review supports major initiatives and policy as outlined in key government documents. For example, the Australian Government's Commonwealth Action Plan seeks to ‘manage the impact of a diagnosis of FASD on the individual and the family to ensure the child and the family are supported through and after the diagnostic process’ (Australian Government, 2014, p. 3). Similarly, the Canadian Government's FASD Framework for Action sets out an objective of “meeting the needs of individuals with FASD, their families and communities” (Public Health Agency of Canada, 2005, p. 9). This includes improving outcomes and helping individuals to reach their full developmental potential. In the US, the National Task Force on Fetal Alcohol Syndrome (US Department of Health and Human Services, 2009) sets out the objectives of intensifying research initiatives around FASD and promoting comprehensive continuums of care for individuals with FASD.

In the 2016/17 Federal Budget the Australian Government committed to providing $10.5M over 4 years to reduce the impact of FASD in Australia (Australian Government, 2016). In 2017 a portion of this funding was allocated to Griffith University, which has brought together a team of experienced academic researchers, clinicians and frontline practitioners. The consortium has been tasked with expanding diagnostic services, interventions and embedding pathways of care in Queensland by 2020. A key customer for which this review is being produced is the consortium, who will use these results in setting direction for intervention policy and pathways of care for FASD in Australia. This evidence will empower the consortium to provide strong return on investment for government, allowing for greater understanding of FASD in young children and further development of evidence‐based treatments to improve the outcomes of these young Australian children and their families.

More broadly, this review will provide evidence that will build the capacity of practitioners and policy‐makers to make informed choices regarding treatment and pathways of care for FASD children globally. This will ultimately drive better outcomes and improve supports as set out in the various government policy agendas.

### 2.4 Objectives

The current review has two aims: (a) systematically gather and synthesize published and unpublished impact evaluations of psychological interventions aimed at improving the executive functioning of children with FASD; (b) data permitting, the review will also provide the first statement of overall treatment effect for EF interventions in children with FASD. The objective is to help practitioners make informed decisions when confronted with FASD clients in real‐life situations. It is also hoped that results will aide policy‐makers tasked with drafting formal public health policy in the area of the impact of alcohol use on citizen health and welfare.

## 3 METHODOLOGY

### 3.1 Criteria for including and excluding studies

#### 3.1.1 Types of study designs

This review will include randomized controlled trials in which participants have been randomly allocated to an experimental or control group and randomized cluster control trials in which pre‐defined clusters of groups are randomized to different conditions. In these designs, the treatment group refers to participants who partake in the intervention designed to improve executive functioning, and the control group refers to those in no‐treatment comparison or treatment‐as‐usual comparison groups. This review will also include quasi‐experimental designs, in which participants are sorted in to pre‐existing groups. In these designs, studies that include a group of participants exposed prenatally to alcohol or with a diagnosis of FASD will be the treatment group, non‐exposed participants will be treated as the control group. Due to the burgeoning nature of this area of research, single group designs that measure pre‐ and post‐outcomes will also be included. These designs are, however, subject to concerns regarding bias and will therefore be analysed separately to RCTs and quasi‐experimental designs (Higgins & Green, 2008).

### 3.2 Types of participants

Participants will comprise children between 3 and 16 years of age who have (a) a formal diagnosis of FAS, FASD, pFAS, ARND or “at risk of FASD” using any of the following diagnostic systems: The Institute of Medicine Diagnostic system (Hoyme et al., 2005), The Washington 4‐Digit Code (Astley & Clarren, 2000), The Canadian Guidelines (Chudley et al., 2005) or the Australian Guidelines (Bower & Elliot, 2016); or (b) classified as having FAS based on facial dysmorphology alone; or (c) confirmed or suspected prenatal alcohol exposure (light, moderate or heavy dosages). Children with a sole diagnosis of ARBD will be excluded, as this condition is not associated with neurological deficits (Lange et al., 2017).

The minimum age range of 3 years has been selected as it is possible to measure EFs at this age. Where the sample includes some children that fall outside of the specified age range, study authors will be contacted and a request made for the data pertaining only to children within the age range. If no response can be obtained, studies will include the children outside of the age range, and sensitivity analyses will be conducted with these studies excluded to determine if inclusion of these studies has impacted the effect estimates.

There will be no geographical restrictions on study location. It is likely that variability exists among different countries/cultures which may produce differences in diagnosis. As such, clear, formal diagnostic criteria will be used for inclusion. Where included studies do not use one of the four formal diagnostic criteria mentioned above (i.e., participants included based solely on confirmed PAE or facial dysmorphology) they will be analyzed separately, allowing for comparison with studies using formal diagnosis.

### 3.3 Types of interventions

Studies will be included if the focus is on improving EFs in children with FASD or related diagnoses (see ‘Description of Intervention’ section). Note that studies where participants receive both psychological interventions and pharmacological interventions concurrently as part of a focal treatment group will be excluded. Comparator groups that will be included are placebo, treatment‐as‐usual, no treatment, alternative treatment (e.g., pharmacological will be included if it is an alternative treatment comparator group), wait‐list control or comparison condition. Studies that report follow up of any length on relevant outcome data will be included. Intervention settings will include Allied Health clinics (e.g., medical/psychological clinics), schools (public or private), pre‐schools or home‐therapy settings.

#### 3.3.1 Types of outcome measures

The review will include outcomes pertaining to the measurement of EF as defined by Diamond (2013). The three core measures of EF will be included in the review (i.e., inhibitory control, working memory and cognitive flexibility) as well as any higher‐order manifestations arising from combinations of core EF abilities (e.g., reasoning, problem solving, planning, attention, etc.). Due to the common use of synonyms in the EF literature, all known synonyms of outcomes listed here will also be included (e.g., ‘self‐regulation’ and ‘behavioural regulation’ will be included under ‘inhibitory control’, ‘shifting’ and ‘flexibility’ will be included under ‘cognitive flexibility’). Where it is unclear whether listed outcomes are synonyms for EFs, authors will be contacted for clarification. Studies will be included if the outcome data are gathered using either standardized measures of EF or parent/teacher reports of EF or related abilities (e.g., attention). Below is a description of standardized assessment measures (not exhaustive) used to measure EF outcome measure in children. There are no additional exclusion criteria applicable to outcome measures. Studies will be included to provide the outcomes reported in the study aligned with the specified conceptualization of executive functions within this document. Note there are no secondary outcomes to be included in the review.

*Tests of Variables of Attention (TOVA).* The TOVA is a neuropsychological assessment that measures attention while screening for ADHD *(*Leark, Dupuy, Greenberg, Corman, & Kindschi, 1996 *). NIH Toolbox* . The NIH Toolbox is an assessment battery which provides measures of a range of cognitive abilities in children aged 3 years+ *(*Gershon et al., 2013 *). A Developmental Neuropsychological Assessment (NEPSY‐II; Attention and EF Domains).* The NEPSY‐II consists of 32 sub‐tests for use in neuropsychological assessment with pre‐schoolers, children and adolescents (Brooks, Sherman, & Strauss, 2009). The following tasks are from the attention or EF sub‐domains: *Behaviour Rating Inventory of Executive Function (BRIEF‐P/BRIEF‐2).* The BRIEF is a standardized psychological assessment tool that measures EFs in pre‐schoolers *(*Gioia, Espy, & Isquith, 2003 *) and children (*Gioia et al., 2003; Gioia, Isquith, Guy, & Kenworthy, 2016 *).*

*Child Behavioural Checklist (CBCL; Attention and ADHD sub‐scales).* The CBCL is a parent‐ or teacher‐completed checklist assessing a broad competencies, adaptive functions and problems in children (Achenbach & Rescorla, 2013).

### 3.4 Duration of follow‐up

Studies with any length of follow‐up will also be included in the review. In order to synthesize data from studies with different lengths of follow‐up, groups will be defined and analysed separately in to Short (0– 3 months), medium (>3 months– 6 months) and long‐term follow‐up (>6 months).

### 3.5 Types of settings

Primary interventions must take place in a health clinic (e.g., medical, psychological clinic) in a school setting or in a home‐therapy setting to be included.

### 3.6 Search strategy

*Search Terms**.*** Search terms have been pilot‐tested and refined in consultation with multiple authors. As FASD was not adequately described prior to 1973, the search will include only studies published after 1972. There will be no restrictions placed on document language or publication status. The terms will be altered as per search functionality within different databases and platforms. Where sources do not allow for advanced searches, a simplified version will be used. The hand search will cover a period of 12‐months prior to execution of the search date to capture any published articles that have not yet been indexed in academic databases. Each search will be recorded in a search record as per current recommended guidelines (Kugley et al., 2016). The terms are presented below in a generic format:

((executive* N/3 (function* OR control* OR atten*)) OR (self N/3 regulat*) OR “effortful control” OR “working memory” OR (cognitive N/3 flexib*) OR (emotion* N/3 (regulat* OR inhibit*)) OR “set shift*” OR reasoning OR planning OR attention OR inhibit*)
AND
((alcohol* N/3 (prenatal* OR fetal OR foetal OR fetus* OR foetus* OR “neurodevelopment disorder*” OR “birth defect” OR “spectrum disorder*”) OR “sentinel facial feature*”)
AND
(RCT OR randomi* OR trial* OR experiment* OR interven* OR therap* OR treat* OR program* OR review* OR “meta‐analy*” OR “meta‐analy*”)
A number of sources will be searched to ensure adequate coverage of the literature. The final search strategy will include key psychological and medical electronic databases, trial registers, grey literature sources and reference lists of relevant studies.

Electronic searches

*Scopus*

• PubMed

• Embase

• PsycINFO

• PsycEXTRA

• CINAHL

• ProQuest Platform: (Dissertations and Theses Global, Family Health, Psychology Journals, Health and Medical Complete, Nursing and Allied Health, Social Services Abstracts)

• Web of Science (Social Science Citation Index, Conference Proceedings Index, Current Contents Connect)

• Cochrane and Campbell Collaboration libraries

*Grey Literature sources*

• Alcohol and Alcohol Science Database (ETOH)

• Alcohol Concern (UK)

• Alcohol and Drug Foundation (Australia)

• National Institute on Alcohol Abuse and Alcoholism

• Foundation for Alcohol Research and Education (FARE)

• The Alcohol Pharmacology Education Partnership

• Australian Centre for Child Protection

• Royal Australasian College of Surgeons

• Society for Research on Child Development (SCRD)

• Social Science Research Network

• Australian Research Alliance for Children and Youth

• NoFASD Australia

• FASDHub Australia

• NoFAS.org

• Russell Family Fetal Alcohol Disorders Association (RFFADA)

• CanFASD (Canada FASD Research Network)

• FASD outreach (British Columbia)

• Fetal Alcohol Network NZ

*Conference proceedings*

• Australasian FASD conference

• International Research Conference on Adolescents and Adults with FASD

• International conference on FASD

*Reference list of relevant papers and other methods*

• Relevant references from all included studies will be harvested

• GoogleScholar will be used to forward citation search all included studies. GoogleScholar has been selected as the platform for citation searching, as studies that may not be published in peer‐reviewed journals are included in citation counts.

• Hand searching the most recent issue of relevant journals: Research in Developmental Disabilities, Developmental Neurorehabilitation, Alcoholism: Clinical and Experimental Research, Child Neuropsychology, and Applied Neuropsychology: Child.

• The following trial registries will be searched: Australia and New Zealand Clinical Trials Registry, ClinicalTrials.gov, Clinical Trials Results, Cochrane Central Register of Controlled Trials (CENTRAL), ISRCTN Registry (controlled‐trials.com), NIH RePORTER, Trials Register of Health Interventions (TRoPHI), UK Clinical Research Network (UKCRN Study Portfolio), WHO International Clinical Trials Registry.

• As a final step authors of relevant studies will be contacted to identify any unpublished studies.

### 3.7 Selection of studies

#### 3.7.1 Title and abstract screening

All studies retrieved using the search strategy will be imported to Endnote for removal of duplicates and eligible document‐types (e.g., blog posts, book reviews). All remaining records will be exported to *SysReview* (Higginson & Neville, 2014). Each record will be subject to title and abstract screening along the following exclusion criteria:

1. Duplicate document
2. Ineligible document type (e.g., book reviews, blog posts, language other than English)
3. Document does not include any topic related to PAE

While efforts will be made to exclude duplicate and ineligible document‐types prior to title and abstract screening, each record will be assessed against these two criteria initially to safeguard against these documents slipping through to screening. Once documents are confirmed unique and eligible‐type, the first content‐relevant criterion will be applied to remove any documents not related to problems relating to PAE.

Following this process all retained records will progress to literature retrieval, where full‐text versions will be located and imported to *SysReview* for full‐text eligibility screening. In the case that full‐text documents cannot be retrieved through existing university pipelines, they will be ordered through university libraries or authors will be contacted for provision of documents.

#### 3.7.2 Full text eligibility screening

All records retained through title and abstract screening will be screened based on the following exclusion criteria:

1. Duplicate document
2. Ineligible document type
3. Document is published before 1973
4. Document does not include topic related to PAE
5. Ineligible participants
6. Ineligible intervention/no intervention
7. Ineligible outcome measure(s)
8. Ineligible methodological design

The first three stages will act as a final check that duplicates, ineligible document‐types and those which do not include topics related to PAE have not progressed through to this stage. A random sample of 10% of documents will be double‐screened blindly and independently by two authors at the title and abstract stage. Independent screening at this stage will continue until an agreement rate of at least 95% is reached, and this rate will be reported in the final review. The final full‐text eligibility screen will be independently completed by two authors, and a third author consulted in the case of disagreement until consensus is achieved.

### 3.8 Data extraction and management

Studies retained following both stages of screening will be coded in *SysReview* as per a standardized coding sheet and the relevant risk of bias tool (see section below). The coding sheet will be pilot‐tested by the research team with a small sample of five articles in the area of child neurodevelopment. Two authors will independently code all eligible studies and discrepancies will be resolved through discussion with a third author until consensus is reached. Studies will be coded along the following broad dimension:

1. Document description (e.g., location, year of publication)
2. Methodological issues (e.g., design, randomization)
3. Participant information (e.g., child diagnosis, descriptives)
4. Intervention (e.g., training/education, setting, duration)
5. Outcomes (e.g., type of EF, measure used, time‐points)
6. Effect size (e.g., type of effect size, how it was obtained)

### 3.9 Assessment of risk of bias in included studies

To ensure valid conclusions the Cochrane Risk of Bias Tool (Higgins, Altman, & Sterne, 2017) will be used to assess risk of bias in studies using RCT design. These studies will be rated as ‘high’, ‘low’ or ‘unclear’ risk of bias along the following domains: sequence generation, blinding of outcome assessors, complete outcome data, selective reporting, and other potential sources of bias (i.e., intervention dose and confounding variables). RCT studies will not be assessed for blinding of participants as the participatory nature of interventions (training and education programs) makes blinding impossible. Where studies do not use random allocation (as in the case of comparing FASD children to typically‐developing children), the Cochrane tool for assessing Risk of Bias in non‐randomized studies of interventions (ROBINS‐I; Sterne et al., 2016) will be used. All studies will be assessed independently against their relevant risk of bias tool. Discrepancies will be discussed with a third author until consensus is achieved. Where studies use a randomized cluster methodology, additional biases will be assessed on the coding form: recruitment bias, baseline imbalances, loss of clusters and incorrect analyses (Deeks, Higgins, & Altman, 2017). Coders will not be made blind to study details during coding (e.g., author names, institutions, journals and results) as some research has suggested little benefit associated with this (Kjaergard, Villumsen, & Gluud, 2001) and both coders are likely to be familiar with a large number of key resources.

### 3.10 Measures of treatment effect

Treatment effect is expected to be expressed in continuous outcome data for all studies. Data will therefore be analysed in terms of standardized mean differences (SMD; the difference between mean values on outcome between experimental and control group, divided by the standard deviation on the outcome of participants). This method is recommended where included studies assess the same outcome (i.e., EF) using a variety of methods (Deeks et al., 2017). Where studies report baseline and postintervention outcome data, SMDs will be calculated using baseline adjusted mean differences (i.e., mean change scores). Standard deviation for the change score will be standardized using the raw standard deviation within groups. In cases where authors do not report standard deviation for change scores, a formula recommended by Lipsey and Wilson (2001) will be used to estimate these figures. No qualitative data will be included in this review.

In the case that studies include only a single group with outcome measures recorded pre‐ and post‐intervention, observations pre‐intervention will be treated as the control group and observations post‐intervention as the treatment group. The effect size will be calculated by treating the data as pre‐post comparison and assessing the difference in means using a *t*‐test. Cohen's *d* effect size will be calculated using a *t*‐statistic and the sample sizes of each observation. The standard error of *d* will then be calculated as the square root of the variance. This is commensurate with procedures proposed by Lipsey and Wilson (2001).

### 3.11 Unit of analysis issues

The unit of analysis for this review will be each study (rather than each report). It is expected that at least some relevant studies will use repeated observations on participants (such as follow‐ups). In these cases several different outcome measures will be defined based on different periods of follow‐up: Short (0–3 months), medium (>3 months–6 months) and long‐term follow‐up (>6 months). These different outcomes will be analysed separately. In cases where studies include multiple types of experimental groups, those which most closely match inclusion criteria will be included, and others excluded (Deeks et al., 2017).

Where a cluster‐randomized methodology is employed, data for individuals are not independent due to dependence within clusters. This means that a unit of analysis error occurs when data are analysed at the individual level. Initially, cluster‐randomized studies will be assessed in terms of appropriate statistical analysis (e.g., multi‐level modelling). In the case that individual participants have been used as the unit of analysis (i.e., inappropriate statistical analysis), the approach recommended by Deeks et al. (2017; Section 16.4.3) will be used to adjust the study's standard error, allowing these studies to be included in the synthesis.

Where there are multiple measures of the same construct reported in a study, conceptually similar outcomes will be grouped to form a composite effect size for that specific executive function using the approach provided by Borenstein, Hedges, Julian, and Rothstein (2009). This composite effect size will then be used in the meta‐analysis**.** For example, in the case that multiple measures assessing cognitive flexibility are included in a single study, data will be combined for each measure into a single cognitive flexibility effect size for that study. Note that direct (standardized/normed neuropsychological assessments) and indirect (e.g. parent self‐report questionnaires) measures of executive will not be combined, even in the case that they measure the same aspect of executive function. This is due to differences in measurement error between these methods (Gross, Deling, Wozniak & Boys, 2015). Thus, direct and indirect outcome measures will be combined and synthesized separately.

### 3.12 Dealing with missing data

The standardized coding form includes information on missing data. In the case that data are missing from documents study authors will be contacted and original data will be sought. Where missing data cannot be recovered, studies will be coded on methods used to cope with missing data and any inherent assumptions made explicit. This data will be included in the final analysis, with the potential impact on findings discussed (Deeks et al., 2017).

### 3.13 Assessment of heterogeneity

This analysis is attempting to synthesis findings from two types of interventions (training and education) and different measures of EFs. It is useful to do this when seeking to offer conclusions about a broader category of intervention and outcomes (i.e., psychological interventions for EFs; Deeks et al., 2017). As such, all studies will initially be analyzed together using a random effects model. Heterogeneity will be examined using the *I* ^2^ statistic, *χ* ^2^ test and *τ* ^2^ (Deeks et al., 2017).

### 3.14 Assessment of reporting biases

In order to ascertain whether publication bias may be influencing results a number of grey literature sources will be searched for relevant material. Key authors will be contacted for unpublished documents relevant to the research question. Websites of key Government and FASD organizations will be examined to find any studies not published in academic journals. Trial registries will also be searched (e.g., http://www.anzctr.org.au) from a range of countries. A funnel plot will be produced to assess whether publication bias is likely to be influencing overall results and, data permitting, subgroup analyses will be conducted to examine whether intervention effects vary by publication source (published versus unpublished).

### 3.15 Data synthesis

After coding each study and extracting/calculating each study's effect size, a random effects inverse variance method (Deeks et al., 2017) will be performed in *Review Manager* (*version 5.3*). This method is used in cases where it is assumed that included studies are estimating different, yet related effects.

### 3.16 Subgroup analysis and investigation of heterogeneity

Data permitting, sub‐group analyses will be conducted to explore whether there is variation in the effects of interventions depending on the following factors:

1. Variation in the number, setting, delivery and intensity of program components;
2. Variation in program participants (e.g., gender, age, comorbid diagnoses, level of PAE); and
3. Variation in the type of EF targeted by the intervention

Meta‐regression will be used as it can include both categorical and continuous variables. It allows for multiple covariates to be explored to explain heterogeneity comprehensively, however can be prone to overfitting when the number of studies included in the analysis is quite small (Houwelingen, Arends, & Stijnen, 2002). Where there is insufficient data to conduct meta‐regression, moderator analysis analogous to ANOVA will be used for categorical factors.

### 3.17 Sensitivity analysis

Data permitting, sensitivity analyses will be conducted to examine a range of factors in the review decision‐making process that may impact the robustness of the meta‐analytic results. These factors include

1. Research designs and risk of bias (e.g., running analyses with only RCTs or studies with low risk of bias)
2. Comparison conditions (e.g., running analyses with different groupings of comparison conditions)
3. Timing of the outcome measure(s)
4. Proportion of study samples with eligible participants meeting inclusion criteria
5. Type of diagnostic assessment or FASD guide utilized

It is not uncommon that additional issues are identified during the review process (Deeks et al., 2017). Where certain decisions are identified to have potential influence on findings, sensitivity analysis will be undertaken by performing analysis with certain subgroups removed. The results of sensitivity analyses will be presented in a summary table.

## ACKNOWLEDGEMENT

This project will be funded under the Drug and Alcohol Program: Fetal Alcohol Spectrum Disorder (FASD) Diagnostic Services and Models of Care Grant Opportunity ‐ H1617G038, Commonwealth Government, Australia.

## CONFLICT Of INTERESTS

Professor Sharon Dawe and Paul Harnett are co‐founders and have been involved in the evaluation of the Parents under Pressure (PuP) program. PuP is a parenting program designed to improve outcomes (including executive function) in high‐risk, substance misusing families, and would therefore qualify for inclusion as an eligible intervention. Both Sharon and Paul have co‐authored a recent systematic review on interventions for improving outcomes in FASD (Reid et al., 2015). To minimize potential bias, other authors will screen and code any papers co‐authored by Professor Dawe or Paul Harnett.

## AUTHOR CONTRIBUTIONS

The review team offers comprehensive coverage of the required skills and experience to successfully produce this review. Joseph Betts (lead reviewer) has experience leading on a range of analytic projects as a statistician for central government (United Kingdom – Department for Business Innovation and Skills). Joseph has previous experience employed at the University of Queensland as a research assistant, aiding in the screening and coding stages of a systematic review project (managed by co‐author Elizabeth Eggins). Joseph's PhD research is focused on exploring new mechanisms of early detection and treatment in FASD populations, and will receive ongoing support from Professor Sharon Dawe as his primary supervisor.

Review tasks will be distributed as such:

• Content: Dawe, Betts, Shelton, Till

• Systematic review methods: Betts, Eggins

• Statistical analysis: Betts, Eggins, Till, Harnett

Information retrieval: Betts, Eggins, Dawe, Chandler‐Mather
